# Assessment of owner-directed aggressive behavioural tendencies of dogs in situations of possession and manipulation

**DOI:** 10.1098/rsos.171040

**Published:** 2017-10-18

**Authors:** Anna Bálint, Gabriella Rieger, Ádám Miklósi, Péter Pongrácz

**Affiliations:** 1 Department of Biology, Lund University, Lund, Sweden; 2 Department of Ethology, Eötvös Loránd University, Budapest, Hungary; 3 MTA-ELTE Comparative Ethology Research Group, Budapest, Hungary

**Keywords:** dog, aggressive behaviour, behaviour test, questionnaire, dog owner

## Abstract

Excessive aggression is a common behaviour problem in dogs that can have various destructive effects on the affected people and the implicated dog. Aggressive behaviour directed towards the owner or other family members is one of the most frequently occurring aggressive phenotypes. Here, we examine the reliability of a short questionnaire assessing aggressive behaviours by two, contextually different behavioural tests: ‘take away bone’ and ‘roll over’. Based on dogs' behaviour in the tests, we sorted dogs (*N* = 93) in two groups for each test, namely a less and a more disobedient/resistant group. The two principal components obtained in our questionnaire—‘obedient’ and ‘aggressive towards owner’—showed significant differences between the behaviour groups. While dogs in the less disobedient/resistant groups had significantly higher ‘obedient’ and significantly lower ‘aggressive towards owner’ scores, dogs in the more disobedient/resistant groups had significantly higher ‘aggressive towards owner’ and significantly lower ‘obedient’ scores. Dogs' age, sex and neuter/spay status expressed their effect through interactions. Males, young dogs and intact dogs were less ‘obedient’ than older ones, while resistant spayed/neutered dogs were more aggressive towards the owner. The questionnaire used is a safe, easy to deploy and time-efficient tool to reliably assess certain owner-directed aggressive tendencies of family dogs.

## 1. Introduction

Dogs represent an integral part of most human societies around the world and fulfil various roles in humans' lives, ranging from being working companions (e.g. police dogs, military dogs and tracking dogs), aiding people as therapeutic animals (e.g. assistance dogs) or being kept as social companions, often being referred to as family members [1,2]. The behaviour of dogs profoundly affects the quality of the relationship between the animal and its social environment, and different behaviour problems can have significant adverse effects on these relations [3]. One of the most serious problems is aggressive behaviour, especially when directed towards human companions, often leading to the disruption of the bond between owners and dogs [4,5]. Injuries caused by aggressive dogs may induce psychological and physical distress on the individual level, and may also represent a problem concerning public health and animal welfare.

Generally, most of a pet dog's social interactions (both affiliative and agonistic) happen within a well-defined human group, usually consisting of the owner/family, relatives and friends (although other animals, companion and livestock, may also participate in a dog's social environment). Reisner [6] found that 35% of dog bites are directed towards friends and neighbours of the family or distant relatives, while 30% are directed to the owner or close family members. Fatjo *et al*. [7] investigated the occurrence of aggressive behaviour in family dogs and found that aggression directed towards owners is the most frequent aggressive phenotype, followed by aggression directed towards unfamiliar dogs and people. The characteristics of the dog–owner relationship also seem to play a crucial role in the occurrence of aggressive behaviour. Several authors concluded that the owner's attitude towards the dog, as well as the amount and the quality of time they spend with the dog or the place where they acquired the dog, may have an effect on the occurrence of aggressive behaviours (e.g. [8–10]).

From an ethological perspective, aggressive behaviour can be defined as a behaviour that serves to resolve conflicts over different resources [11,12]. It may occur within or between species (intra- and interspecific aggression), and according to a number of different authors, aggressive behaviour is usually categorized into several different types, as for example: possessive, defensive, dominance and sexual aggression [3,13–15]. Although the precise definition of each category is often disputed, some authors found that owner-directed aggressive behaviour was associated with fear, impulse control or possessive aggression [3,16–19]. The accurate assessment of aggressive behaviour is important to improve the quality of the dog–human companionship and to address important animal welfare and community health issues. Because of its context specificity and possible ethical consequences, it is often very difficult to observe aggression to humans in an experimental setting, and even laboratory tests often fail to deliver conclusive results [20]. A number of methods have been developed to measure the behaviour of dogs, some of which are based on the direct observation of dogs' response to different test situations (e.g. [21,22]). However, the evaluation of aggressive behaviour is especially difficult by behavioural testing, with many owners and dog handlers being reluctant to participate in these tests because of the dog's known or anticipated aggressive behaviour. From an animal welfare perspective, exposing dogs to situations which might make them react aggressively can put them at risk and may aggravate the aggressive tendencies of certain individuals. From an experimental point of view, collecting data from a sequence of tests could be subject to order effect (e.g. habituation and sensitization) [23,24], potentially leading to biased results. Even under controlled conditions, environmental and personnel differences may affect testing. Also, because aggressive behaviour has high context and stimulus specificity [3], it is often difficult to draw general conclusions from certain tests or test batteries. For example, if the behaviour test is conducted by an experimenter that is a stranger to the dog, the results are not necessarily directly transferable to a situation where the owner interacts with the dog. Nevertheless, if conducted and interpreted carefully, direct behavioural observations are very important and indispensable tools in the assessment of aggressive behaviour, especially in a general population of dogs, where aggressive tendencies may have not emerged at home yet, unlike in a clinical population with already known incidences of aggression.

One other well-known way to collect information on the behaviour of dogs is by means of questionnaires [2,20,25]. Questionnaires have several advantages, for example they can easily target a variety of topics and can be used on various different subjects, while enabling the relatively easy collection of extensive datasets. Moreover, if well-constructed, data analysis can be fast and convenient [26]. However, some disadvantages of questionnaire studies should also be taken into account. Various biases can arise from the diversity of the sampled human populations, the owners' willingness to cooperate or because of preconceptions of the behaviour of different dog breeds. Misinterpreted causal relationships between certain variables can also be a source of the distortion of results [2, pp. 60–63].

To determine how accurately a questionnaire predicts the behavioural phenotype in question, it is highly important to establish its validity [27,28]. This, for example, can be established by comparing the results of the questionnaire assessment to the behaviour shown in contextually relevant behaviour tests.

In this study, our aim was to develop a questionnaire which could accurately assess the owner-directed aggressive behavioural tendencies of dogs. To validate the questionnaire, the subject dogs also participated in two, contextually relevant behaviour tests that can often be encountered in the everyday life of dog owners. In one test, the owners were asked to take away a bone from their dog (take away bone), while in the other one, they were asked to physically manipulate the dog to a position of dorsal recumbence for 30 s (roll over), without using any verbal commands or body signals (see the exact details below in Experimental procedure). The ‘roll over’ test did not include any intimidation or excess forcing of the animal, and owners were allowed to quit the test if they considered that the dog was getting overtly stressed while lying on its back. While the rationale for using the ‘take away bone’ test as a gauge of owner-directed aggression might be straightforward, the reason to use the ‘roll over’ test may be less obvious. However, because the physical manipulation or restraint of the dog is often inevitable (e.g. at the veterinary's office and at a dog park if the dog needs to be held back), the manoeuvre involved in the ‘roll over’ test may indeed serve as a contextually relevant paradigm.

The questionnaire ratings were statistically compared with the behavioural scores of the behaviour tests. Our hypothesis was that if the questionnaire can discriminate between dogs exhibiting more or less owner-directed aggression, then these subjects would also show more aggressive responses in the behaviour tests, thereby demonstrating the predictive value of the questionnaire.

## 2. Material and methods

### 2.1. Subjects

The subjects (*N* = 93, 43 males and 50 females) were recruited from the attendees of four different Hungarian dog schools and two additional dog training summer camps on a voluntary basis. All dogs were older than 10 months. The selection of subjects was regardless of their history of aggressive behaviour—in other words, having a history of biting was neither a requirement, nor did the experimenters ask about this history at the time of the tests. There was no special requirement regarding dogs' level of training experience. The list of participants and their basic descriptive information (breed, age and neuter/spay status) are indicated in table 1.

**Table 1.** List of subjects, with their breed, sex, neuter/spay status (0 = intact; 1 = neutered/spayed) and age (in years). Owner's sex means the sex of the person who performed the ‘take away bone’ test and ‘roll over’ test with the dog.

dog's no.	breed	dog's sex	neuter/spay	age	owner's sex
1	mixed breed	male	0	5	female
2	Golden Retriever	female	0	2	female
3	Boxer	female	1	8	male
4	Boxer	female	1	1	female
5	Golden Retriever	female	0	1	female
6	Border Collie	male	0	1	female
7	Dogo Canario	male	1	4	female
8	Border Collie	male	1	2	female
9	Golden Retriever	male	0	1	female
10	Vizsla	male	0	1	male
11	Dobermann	male	1	7	female
12	Dog de Bordeaux	male	0	2	female
13	Bichon Havanese	male	1	2	female
14	Golden Retriever	male	0	6	female
15	Pumi	female	0	7	female
16	French Bulldog	male	0	1	female
17	mixed breed	female	1	8	female
18	Boxer	female	1	3	female
19	Golden Retriever	male	1	2	female
20	mixed breed	female	1	2	female
21	Vizsla	male	1	3	female
22	Dachshund	male	0	7	female
23	Yorkshire Terrier	female	0	2	female
24	mixed breed	female	1	9	female
25	Dogo Argentino	male	1	1	female
26	Groenendael	female	0	1	female
27	Brussels Griffon	male	0	1	female
28	German Shepherd dog	female	1	7	male
29	Pumi	female	0	8	female
30	English Cocker Spaniel	female	0	8	female
31	mixed breed	female	0	1	female
32	Pumi	female	0	4	female
33	Vizsla	female	1	2	female
34	Sarplaninac	male	0	5	male
35	mixed breed	male	1	7	female
36	Airedale Terrier	female	0	2	female
37	Rottweiler	female	0	3	female
38	Border Collie	male	0	2	female
39	Golden Retriever	female	1	4	male
40	mixed breed	female	1	2	female
41	Vizsla	male	1	7	female
42	Border Collie	male	0	1	female
43	Westie	female	0	2	female
44	mixed breed	female	1	12	female
45	mixed breed	male	0	2	male
46	Parson Russell Terrier	female	0	2	female
47	German Shepherd dog	female	0	1	male
48	Rottweiler	female	0	5	female
49	Golden Retriever	male	0	2	female
50	Westie	female	0	1	female
51	Labrador Retriever	male	0	1	female
52	Cav. K. Ch. Spaniel	female	1	1	female
53	Labrador Retriever	male	0	4	female
54	Welsh Terrier	female	1	1	male
55	Groenendael	female	0		female
56	mixed breed	female	1	1	female
57	Vizsla	female	1	7	male
58	Fox Terrier	female	0	4	female
59	mixed breed	female	0	4	female
60	Husky	female	0	1	female
61	Beagle	male	0	7	male
62	Boxer	male	0	1	female
63	mixed breed	female	1	2	female
64	Vizsla	female	0	6	female
65	Dachshund	male	0	3	female
66	mixed breed	female	0	2	female
67	Beagle	female	0	1	female
68	Beagle	female	1	1	female
69	English Cocker Spaniel	male	0	2	female
70	Rhodesian Ridgeback	male	0	1	male
71	Vizsla	female	0	10	female
72	English Cocker Spaniel	female	1	5	female
73	Dachshund	male	1	8	female
74	German Shepherd dog	male	0	4	female
75	Am. Staff. Terrier	female	1	6	female
76	Pumi	female	1	11	female
77	Beagle	male	0	1	female
78	Gordon Setter	male	0	1	male
79	Vizsla	female	1	2	female
80	mixed breed	female	0	1	female
81	Westie	male	0	2	female
82	Jack Russel Terrier	male	0	1	female
83	mixed breed	male	0	1	male
84	mixed breed	male	1	8	female
85	Pumi	male	0	1	female
86	Vizsla	female	1	5	female
87	Am. Staff. Terrier	female	1	2	female
88	Parson Russell Terrier	male	0	4	female
89	Dachshund	male	1	8	female
90	Golden Retriever	female	1	2	female
91	Border Collie	male	0	1	female
92	mixed breed	male	0	5	female
93	Parson Russell Terrier	female	0	2	female

### 2.2. Experimental procedure

Behaviour tests were performed outdoors, in secluded areas of dog schools where only the owner and the experimenter (G.R.) were present with the subject. All tests were recorded by video cameras, and variables were assessed and extracted from the video footage later. First, the owners completed the questionnaire (see appendix A), after which they participated in the two behaviour tests: the so-called ‘take away bone’ and ‘roll over’ tests. According to Netto & Planta [25], consecutive experimental trials testing the different aspects of aggressive behaviour in dogs might increase the animals' aggressive tendencies. To reduce risk of this effect, we waited 2–3 min between the behaviour tests. For each subject, we conducted the tests in a fixed order: questionnaire—take away bone—roll over. The questionnaire was done first because we did not want owners to be biased by the fresh experiences with their dogs' responses in the behaviour tests. We left the ‘roll over’ test to the last, because this test potentially can be stressful for the dogs; therefore, if the ‘take away bone’ test would follow consecutively, some dogs may be reluctant to chew on the bone.

Before both tests, the experimenter first carefully explained and demonstrated the procedure to the owner. Owners were also informed that they could stop the experiment at any point if they felt that the procedure was causing an unacceptable level of stress to their dog or risk of injury to them.

### 2.3. Questionnaire

Our questionnaire consisted of 20 items concerning different aspects of the dogs' aggressive behavioural tendencies and the dog–owner relationship (see appendix A). Certain questions (2, 3, 4, 6, 10, 11, 13, 16, 17, 18, 19) are adopted from a study investigating dog personality traits [29], with some modifications implemented to address dog–owner interactions specifically. All other items were formulated for this study.

The owners had to decide how the statement or description of the given item applied to their dog and had to indicate this on a printed response scale ranging from 0 to 10 cm, where 0 meant ‘never’ and 10 meant ‘always'. The questionnaire was handed out to the owners at the testing site, and no further assistance was provided for the owners for its completion. The questionnaires were completed prior to the behaviour tests; however, the experimenters did not evaluate them before the tests, avoiding the formation of expectations about the dogs' upcoming reactions.

### 2.4. *‘*Take away bone’ test

In this test, the dog was tethered to a tree or fence pole by a 2 m long, sturdy rope (for detailed method see [24,30]). On one arm, the owner wore an artificial hand (casted from gypsum and covered with a cloth-glove, providing a life-like appearance to it), while also wearing a pressed paper forearm-guard under his/her sleeve, for further protection. In the other hand, the owner held a large, cooked bone, tied on a string. The bone (preferably pork) was large enough that even the bigger dogs could not devour or chew it apart during the test. We used a new bone on each separate day of testing; however, we did not switch bones between subjects on the same day. Over the course of the test, all participants were standing outside the range of the rope (i.e. outside the range of a potential bite from the dog).

The owner gave the dog the bone and encouraged it to take it. In case the dog moved away with the bone (out of reach) from the owner during the test, the owner tried to readjust its position by pulling the bone on the string.
After the dog had taken the bone and had chewed on it for approximately 30 s, the owner stepped closer to the dog and patted the dog's back three times with the artificial hand.
After patting the dog, the owner reached for the bone, asking the dog to release it by using a verbal command only once (e.g. ‘Release!’ or ‘Give it to me!’ depending on what the particular owner used to say in similar situations). From this time on, the owner was not allowed to talk to the dog.
The owner put the artificial hand on the bone and left it there for 5 s.
If the dog had not already let go of the bone, the owner started to take the bone away by pulling the string with one hand, while keeping the artificial hand on the bone. The test was continued even if the dog was snarling or growling, and it was only ceased if the dog let go of the bone, or if the dog made an attempt to attack the owner. We instructed the owners to pull away the bone without hesitation with a slow but continuous pace, and as the dogs seldom expressed any ongoing resistance (e.g. grabbing the bone), the test ended within a minute. In those cases when the dog tried to bite the artificial hand, the test was immediately terminated.

The test started when the owner first patted the dog's back and lasted for 1 min.

### 2.5. ‘Roll over’ test

Preceding the test, the owner took the dog on leash and put a muzzle on it adjusted to its head-size. All dogs were familiar with wearing a muzzle, as in Hungary it is mandatory to muzzle any dog that is travelling by public transportation; or depending on the regulations of the township, muzzling can be mandatory even for dogs that are taken to public areas.

The dog was tethered again to a tree or a fence pole by a 2 m long, sturdy leash.

The owner gently tried to make the dog lay down on its back (so that dogs' legs do not touch the ground), without using any direct commands or body signals. Only calming words or patting was allowed.

The owner attempted to keep the dog in this position for 30 s; the overall duration might consist of separate, shorter periods in the case that the dog got up from lying during the test. The owner was instructed not to force the dog physically to lay on its back, but (s)he was allowed to hold it gently even if the dog tried to stand up.

The test started at the owner's first attempt to lay the dog down and lasted for a maximum of 2 min.

### 2.6. Statistical analysis

Measured variables in the behavioural tests:

‘Take away bone’ test

Removal of the bone (0–4)

0:The dog releases the bone during the first pat on its back.
1:The dog releases the bone when the owner reaches for it/asks for it.
2:The dog releases the bone if the artificial hand rests on it.
3:The dog only releases the bone after tugging.
4:The dog does not release the bone at all.

Dogs with scores 0, 1, 2 were assigned to the ‘Tractable’ group (*n* = 33). Dogs with scores 3 or 4 were sorted to the ‘Possessive’ group (*n* = 61).

‘Roll over’ test

Resistant behaviour (0–3)

0:The dog does not show any resistance.
1:The dog resists only once, but after that it can be laid easily on its back.
2:The dog resists more than once, but eventually can be laid on its back.
3:The dog resists throughout the procedure (cannot be laid on its back).

Dogs with score 0 or 1 were assigned to the ‘Compliant’ group (*n* = 60), while dogs with score 2 or 3 were sorted to the ‘Resistant’ group (*n* = 34).

Since we had a large set of variables (20 items from the questionnaire), we performed a principal component analysis (PCA) based on correlations between variables with Varimax Rotation. The number of PCA components was chosen using the break point of the Scree plot [31]. For further simplification of the components, we applied a backward elimination approach, excluding step-by-step those parameters that had low loading (less than 0.5) or contributed to more than one component with similar absolute loading. Cronbach's *α* was calculated to assess the internal consistency of the final extracted factors and for testing the repeatability of the measurement [32].

Regarding the behaviour tests, an independent coder reanalysed 12 randomly chosen videos for reliability testing. The behavioural scoring of the two coders (Pearson's correlation, removal of the bone: *r* = 0.892; *p* < 0.001; resistant behaviour: *r* = 0.86; *p* < 0.001) showed strong correlation; thus, we accepted the coding to be reliable. Based on their behaviour in the take away bone and ‘roll over’ tests, dogs were sorted in behavioural groups indicating their disobedient/resistant behavioural tendencies. To validate the questionnaire, we compared dogs' questionnaire scores (the scores of the components resulting from the performed PCA) against dogs' behavioural scores in the different behavioural groups. We used general linear model (GLM) analysis, where besides the groups from the two behavioural tests we also included sex, neuter/spay status and age of dogs in the models as independent variables. For this, dogs were sorted to three groups of age (below 2 years of age (*N* = 29); 2–6 years (*N* = 43) and over 6 years (*N* = 21)). We performed back-step model selection to eliminate the non-significant interactions. Results of the final models are reported. Where it was applicable, Tukey's *post hoc* tests were performed to reveal between-group differences.

### 2.7. Experimental groups

Based on their behavioural scores in each test, dogs were sorted into two groups, indicating their disobedient/resistant behaviour. Therefore, we had a more and a less disobedient/resistant group according to each behaviour test. Based on the ‘take away bone’ test, these groups were ‘possessive’ (‘disobedient/resistant’, the bone could only be removed by tugging or not at all) and ‘tractable’ (‘less disobedient/resistant’, the bone could be removed by patting the dog, reaching for or touching the bone). Based on the ‘roll over’ test, the resulting groups were ‘resistant’ (‘disobedient/resistant’, dogs that constantly tried to escape and showed considerable struggle) and ‘compliant’ (‘disobedient/resistant’, dogs did not show escape behaviour or only made feeble attempts).

## 3. Results

### 3.1. Principal component analysis on the questionnaires

The PCA resulted in three components, based on eight questions. Based on the corresponding items, these components were labelled as ‘obedient’, ‘aggressive towards owner’ and ‘barking’. After examining the consistency of the components, only the ‘obedient’ (Cronbach's *α*: 0.812) and ‘aggressive towards owner’ (Cronbach's *α*: 0.688) components seemed to be consistent, while the ‘barking’ component appeared to be inconsistent (Cronbach's *α*: 0.41). Therefore, we only used the first two components in our further analysis (table 2).

**Table 2.** Results of principal component analysis.

questions	obedient	aggressive towards owner
The dog can be called back even if there are other dogs, animals or humans in its vicinity	0.907	
Sometimes the dog's attention is so distracted, that it impairs its obedience^a^	0.856	
The owner can easily end unwanted activities (e.g. by verbal inhibition)	0.765	
The dog growls when being groomed, bathed or when the paws/ears are being cleaned		0.860
The dog responds threateningly/shows intimidating behaviour if being punished or disciplined		0.837
If being disturbed while resting, the dog growls or snaps		0.639
Cronbach's *α* coefficient	0.812	0.688
explained variation	34.30%	13.75%

^a^This question was scored inversely, that is, the dogs with higher scores were those whose obedience could not be interrupted by the dog's distracted attention.

We analysed the correlation between the scores of the two components (obedient versus aggressive towards owner). We found a weak but significant negative correlation (Pearson's *r* = −0.265; *p* = 0.01; *N* = 94).

Table 2 summarizes the questions belonging to the two consistent components emerging after the PCA. Loadings of the different questions corresponding to each component, the Cronbach's *α* values and the explained variation values are also included.

### 3.2. Controlling for the predictive value of the questionnaire by the behavioural tests

After performing the behavioural tests, 30 dogs proved to be ‘less disobedient/resistant’ in both tests (compliant/tractable), 17 dogs were ‘more disobedient/resistant’ in both tests (resistant/possessive), 38 dogs were ‘more disobedient/resistant’ in the ‘roll over’ test but ‘less disobedient/resistant’ in the ‘take away bone’ test (resistant/tractable), and finally, only eight dogs were ‘less disobedient/resistant’ in the ‘roll over’, but ‘more disobedient/resistant’ in the ‘take away bone’ test. We analysed the contingency of these data with Fisher's exact test that showed no significant association (*p* = 0.35).

In the GLM analysis, we compared dogs' component scores (obedient and aggressive towards owner) against the behavioural scores of the two sets of behavioural groups (tractable–possessive and compliant–resistant). That is, we examined whether the ‘obedient’ scores are higher and the ‘aggressive towards owner’ scores are lower for those dogs that are in the ‘less disobedient/resistant’ (tractable and compliant) than for those in the ‘more disobedient/resistant’ (possessive and resistant) groups.

**Figure 1.**
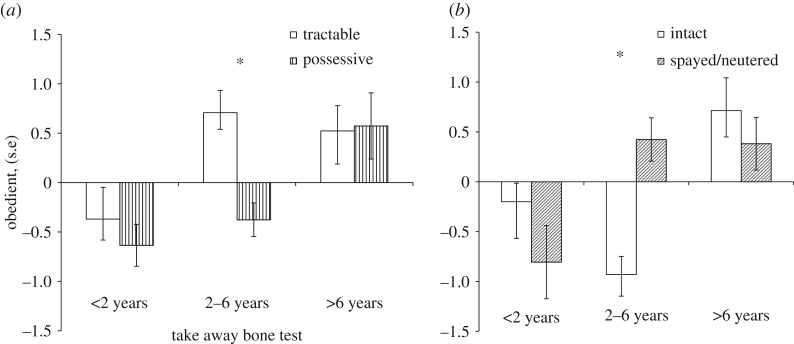
(*a*) Comparison of ‘obedient’ scores of dogs between groups based on dogs' age and the results of the ‘take away bone’ test. After the behavioural test, dogs were sorted to ‘less aggressive’ (tractable) and ‘more aggressive’ (possessive) categories. We found a significant interaction between age and behavioural category. Bars and whiskers represent mean ± s.e. GLM with Tukey's *post hoc* test, *p < 0.05. (*b*) Comparison of ‘obedient’ scores of dogs between groups based on dogs' age and neuter/spay status, after a significant interaction was found between these two factors. Bars and whiskers represent mean ± s.e. GLM with Tukey's *post hoc* test, *p < 0.05.

Our results showed that the ‘obedient’ scores were significantly affected by the sex (*F*_1,82_ = 5.482; *p* = 0.022) and the age (*F*_2,82_ = 6.270; *p* = 0.003) of the dog and, importantly, also by the behavioural group (*F*_1,82_ = 4.891; *p* = 0.030) in the ‘take away bone’ test. Behaviour type in the ‘roll over’ test (*F*_1,82_ = 0.178; *p* = 0.674) and the neuter/spay status of the dog did not have a significant effect on the ‘obedient’ scores (*F*_1,82_ = 0.457; *p* = 0.501). We also found significant interaction between the age and neuter/spay status (*F*_2,82_ = 3.388; *p* = 0.039); and also between the age and behavioural group in the ‘take away bone’ test (*F*_2,82_ = 3.468; *p* = 0.036). According to the *post hoc* analysis, dogs over 6 years of age showed high and the youngest dogs showed low ‘obedient’ scores, independently of their behavioural type in the ‘take away bone’ test, although dogs of the youngest age group had a tendency to be more obedient in the ‘tractable’ group. However, the ‘obedient’ scores of younger adult dogs (between 2 and 6 years of age) showed a strong effect of the behavioural type: ‘possessive’ dogs had significantly lower ‘obedient’ scores than ‘tractable’ dogs (figure 1*a*). Regarding the interaction between dogs' age and neuter/spay status, while older dogs showed again the higher ‘obedient’ scores compared with the youngest dogs, dogs between 2 and 6 years of age showed an interesting difference based on their neuter/spay status: intact dogs were significantly less ‘obedient’ than the neutered/spayed ones (figure 1*b*). Finally, female dogs had significantly higher ‘obedient’ scores than the males did.

**Figure 2.**
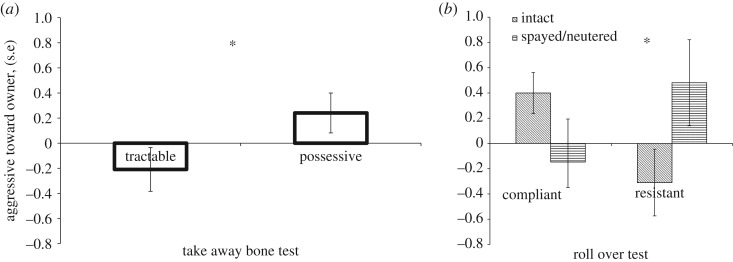
(*a*) Comparison of ‘aggressive towards owner’ scores of dogs between groups based on the results of the ‘take away bone’ test. After the behavioural test, dogs were sorted to ‘less aggressive’ (tractable) and ‘more aggressive’ (possessive) categories. Bars and whiskers represent mean ± s.e. GLM with Tukey's *post hoc* test, *p < 0.05. (*b*) Comparison of ‘aggressive towards owner’ scores of dogs between groups based on the neuter/spay status of dogs and the results of the ‘roll over’ test. After the behavioural test, dogs were sorted to ‘less aggressive’ (compliant) and ‘more aggressive’ (resistant) categories. Spay/neuter status and behavioural category are in significant interaction with each other. Bars and whiskers represent mean ± s.e. GLM with Tukey's *post hoc* test, *p < 0.05.

The ‘aggressive towards owner’ scores showed only one main effect: the behaviour type in the ‘take away bone’ test (*F*_1,85_ = 4.313; *p* = 0.041). Neither of the other factors had significant effect: behaviour group in the ‘roll over’ test (*F*_1,85_ = 0.300; *p* = 0.585); sex (*F*_1,85_ = 3.179; *p* = 0.078); age (*F*_2,85_ = 0.025; *p* = 0.975) and neuter/spay status (*F*_1,85_ = 1.449; *p* = 0.232). However, we found a marginally significant interaction between the behaviour type by the ‘roll over’ test and neuter/spay status of dogs (*F*_2,85_ = 3.882; *p* = 0.052). The *post hoc* analysis showed that ‘possessive’ dogs had significantly higher ‘aggressive towards owner’ scores than ‘tractable’ dogs (figure 2*a*). According to the interaction, ‘compliant’ dogs had rather similar scores of aggression, independently of their neuter status. However, while the intact ‘resistant’ dogs showed the lowest ‘aggressive towards owner’ scores, spayed/neutered ‘resistant’ dogs had the highest scores of aggression (figure 2*b*).

## 4. Discussion

In this study, our aim was to develop a reliable questionnaire designed to assess certain aggressive tendencies of dogs, by validating it with two, contextually different behaviour tests. After analysing the correlation pattern of the results of the questionnaire, we extracted two principal components: ‘obedient’ and ‘aggressive towards owner’. Based on their behaviour shown in the behaviour tests, dogs were sorted into two groups in each behaviour test, one including dogs exhibiting more (possessive and resistant) and one including dogs exhibiting less disobedient/resistant responses (tractable and compliant). Comparing the principal component scores of dogs in the more and less disobedient/resistant behaviour groups, we found that especially the ‘take away bone’ test gave a congruent result with the questionnaire, as dogs in the ‘possessive’ group had significantly higher ‘aggressive towards owner’ and significantly lower ‘obedient’ scores than those in the ‘tractable' group. Dogs' age, sex and neuter/spay status also played important roles. Young dogs showed, in general, low obedience, whereas the oldest age group had invariably high ‘obedient’ scores. In the ‘take away bone’ test, the above mentioned effect of dogs' behaviour on the obedience was the clearest, however, in the young adult age group. Females and neutered/spayed dogs also showed higher obedience. The other principal component from the questionnaire (aggressive towards owner) was solely affected by the behaviour type in the ‘take away bone’ test, as no age, sex or neutering/spaying effect was found. Dogs' behaviour in the ‘roll over’ test made a difference only in the case of the ‘aggressive towards owner’ trait—we found an interaction with neuter/spay status. According to this, those intact dogs that showed strong resistance in the test were otherwise less aggressive by the owners' opinion than the neutered/spayed ‘resistant’ dogs.

From the complex results, the effect of dogs' age is in line with earlier findings, describing older dogs as more calm [33]; meanwhile, the accumulating effect of training and daily structured interactions can also cause older dogs to seem to be more ‘obedient’ for the owner. Neutering/spaying has reportedly an ambiguous effect on the occurrence of problems with different types of aggression in dogs (e.g. [34,35]). For example, while neutering seems to lessen these problem behaviours in males, spayed females are more often reported as having problems with aggression [36].

Our results are important from several perspectives. Although there are a number of behaviour tests aimed at evaluating and determining the aggressive behaviours of dogs, these tests also have their limitations. Validated questionnaires are certainly of great value, not only because of their convenience, but also because they offer an adequate alternative to avoid the potential pitfalls of behavioural testing.

In recent years, a number of different questionnaires have been developed in order to determine the aggressive tendencies of dogs (e.g. [35]). Probably the most widely used large-scale survey is the C-BARQ [37] that, among other behavioural features, also targets aggressive behaviours such as dog-, stranger- and owner-directed aggression. The applicability of the C-BARQ (which was developed on the population of subjects in North America) was proved in other countries (e.g. Japan [38]), and also in more specific investigations, targeting for example particular breeds (e.g. in Golden Retrievers [39]). The short survey we propose in this study adds to the results obtained earlier by the C-BARQ from the aspect that here we empirically tested owner-directed aggression and disobedience on a population of volunteering dog–owner dyads. Our sample was not specifically biased towards dogs where behavioural problems (i.e. biting history) were prevalent—on the contrary, we approached the ‘typical’ clientele of an average dog school. By comparing the dogs' responses from the two behavioural tests with the answers of their owners to the questions in the survey, we could directly assess the construct validity of our questionnaire—an issue that was originally handled in the case of the C-BARQ [37] by comparing the results of the ‘average’ responders with the answers of owners of dogs with behavioural problems. Although some questionnaires seem to provide a reliable assessment of aggressive behaviour (e.g. [22]), many studies failed to find consistency between behaviour test results and questionnaire outcomes, or found correlations only between some aspects of the dog's behaviour and questionnaire ratings (e.g. [40,41]). Unfortunately, in some cases, aggressive behaviour (e.g. manifested in biting history) directed towards the owner or family members remains undetected (e.g. [24]), although this is a crucial aspect of the dog's behaviour. Marder *et al*. [30] assessed the aggressive tendencies of shelter dogs (with, among others, the ‘take away bone’ method) and compared the results with the later experiences of adopting owners. Although their study revealed a considerable number of false positives, the method they used can still be considered as mostly reliable in detecting potentially risky dogs. Our controlled questionnaire offers a reliable method to assess some aspects of dogs' aggressive behaviour shown towards their owners in a population of average companion dogs.

It is worth noting that many (46 out of 93) dogs that were categorized as acting less disobediently/ resistant in one behaviour test (tractable and compliant groups) showed a more disobedient/resistant behaviour (possessive and resistant) in the other test. This discrepancy in the behaviour of dogs could be partly due to methodological differences. Since there is more opportunity for individual differences to appear during the ‘roll over’ test (less rigorously controlled procedure and more human contact), the less consistent technique may have resulted in more variable responses from dogs. Apart from the possible effect of the methodology itself, this phenomenon (i.e. no perfect overlap between the subjects found to be ‘possessive’ and ‘resistant’) should not be very surprising, because aggressive behaviour can be rather context or stimulus specific, and might even change/escalate rapidly [3]. It was found recently that different forms of human-directed aggressive behaviours (against strangers on or outside of the property and against family members) rarely coincide in the same dog, and they can be affected by human- and dog-related factors such as the owner's age, breed of the dog or the training techniques the particular dog was subjected to [42].

The fact that high ‘obedient’ scores coincide with low ‘aggressive towards owner’ scores in the ‘less obedient/resistant’ behaviour groups (and vice versa) may point to an interesting relationship between the traits underlying the two components. In line with our findings, several authors have also found that less aggressive dogs obey more readily (e.g. [8,43,44]), and they concluded that this was linked to the characteristics of the dog–owner relationship. In this sense, although obedience and aggressive behaviour are qualitatively different aspects of behaviour, under certain circumstances they can indicate the quality of the dog–owner relationship.

In our study, the reactions given to certain situations—such as taking the dog's food away or physically manipulating it—could also be affected by the dog's prior experiences, training, breed-specific traits and state of neutering (e.g. [45]). According to Guy *et al.* [45], more than 40% of dogs with ‘worst bite incident’ showed possessive aggression according to the owners. This type of aggression may also involve possessive behaviour with food, and there is ample evidence that the ‘take away bone’ test has a positive predictive value in this regard [24,46]. However, the ‘roll over’ technique has a more debated usefulness not only as part of the dog trainer's toolkit (e.g. [47]), but also as a reliable predictor of the dog's aggressive behaviour. Klausz *et al*. [24] found that dogs with known history as ‘biters’ did not show more aggression or struggling during the ‘roll over’ test than ‘non-biter’ dogs. It is worth noting that the history of actual biting incidents was not included in our study. In our study, the ‘roll over’ test gave less consistent results compared with the ‘take away bone’ test, as the behavioural groups derived from the ‘roll over’ test had an effect only in the case of the ‘aggressive towards owner’ components. However, as this result (in interaction with the neuter/spay status of the subjects) is still congruent with the questionnaire (‘resistant’ dogs had the highest ‘aggressive towards owner’ scores), we may also regard the ‘roll over’ test as predictive somewhat to the aggressive behaviour of a dog towards its owner. The reason for the seemingly different results in our study and that of Klausz *et al*. [24] could be that while they were concentrating on dogs with known biting incidents, in our case extreme incidents of owner-directed aggression were not set as prerequisites for participating in the study. In the typical companion dog population, being rolled and restrained on the back may have a differential effect. In those dogs that have a positive relationship with their owners, the procedure may have elicited minimal to no struggle, while in those dogs that scored high on particular items of our questionnaire, being restrained on their backs may have triggered fear or frustration-related responses [48]. The robustness of the questionnaire used in our study is underlined by the fact that the two components resulting from the owners' answers were in significant agreement with both otherwise considerably different behavioural tests.

## 5. Conclusion

In this study, we successfully developed a short questionnaire that can reliably assess the aggressive behavioural tendencies of dogs against their owners, without being subjected to the potential risks of direct behavioural testing. Thereby, the assessment of aggressive behaviour becomes possible in those dogs that are otherwise often precluded from participating in behaviour measurements because of their aggressive propensities. By controlling the questionnaire with two relevant behaviour tests, we showed that it could serve as a versatile and reliable research tool for the quick assessment of owner-directed aggressive tendencies in dogs. In the future, it would be interesting to widen the applicability of our questionnaire, also by testing its predictability in other behavioural situations. In the case of subjects living at dog shelters, before offering dogs for adoption, the questionnaire would be applicable only after a longer period when the caretakers gained enough personal experiences about particular dogs through their everyday interactions.

This would allow us to undertake measurements in a much a wider population, providing not only researchers but also dog owners and handlers with valuable and relevant information.

## Supplementary Material

Raw Data

## Data accessibility

All data are available in the electronic supplementary material.

## Appendix A

The 20 questions used in this study for evaluating the owner-directed aggressive tendencies of dogs are listed below. To complete the questionnaire, owners had to indicate their answer (by putting a mark) on a 10 cm long printed ruler, where 0 signifies ‘Never’ and 10 signifies ‘Always’.

The dog can be called back even if there are other dogs, animals or humans in its vicinity.
The owner can easily stop unwanted activities (e.g. by verbal inhibition).
Sometimes, the dog becomes so overactive during play that the activity has to be ended.
The dog intensely defends its food, ball or other assets, even from the owner.
The dog has a skill to seek out and steal food from anywhere, sometimes even from the hands of people.
The dog demands physical contact with the owner: it often cuddles or snuggles up to the owner or leans its head in the owner's lap.
The dog growls when being groomed, bathed or when the paws/ears are being cleaned.
If being disturbed while resting, the dog growls or snaps.
The dog seizes every opportunity to escape and run away, and after successfully getting away, it is very difficult to call him back.
The dog follows the owner whenever and wherever it is possible.
The dog might bite or snap at others (humans or dogs) in the presence of the owner.
The dog responds by barking or growling to situations/events it does not appreciate or opposes.
The dog responds threateningly/shows intimidating behaviour if being punished or disciplined.
The dog is highly frustrated when left alone, continuously barks or shows destructive behaviour.
If the dog wants to obtain something, it pursues that persistently or even aggressively.
The dog behaves in an assertive manner.
If the dog once understands that something is forbidden, it is easy to prevent the same thing on a subsequent occasion.
Sometimes, the dog's attention is so distracted that it impairs its obedience.
The dog often barks in unusual or novel situations. In these cases, it is almost impossible to calm it.
During clicker training, the dog is usually trained by the so-called shaping method.
